# HBV-miR-3 is closely related to HBV replication and strongly predictive of HBeAg seroconversion in PegIFN-α treated patients

**DOI:** 10.1038/s41598-024-52060-0

**Published:** 2024-01-17

**Authors:** Zhenyu Xu, Yun Xu, Zhenyu Wu, Sujuan Wang, Min Zhang, Yongfang Jiang, Guozhong Gong

**Affiliations:** 1 Department of Infectious Diseases, The Second Xiangya Hospital of Central South University, Changsha, 410011 Hunan China; 2 FuRong Laboratory, Changsha, Hunan China; 3 Clinical Medical Research Center for Viral Hepatitis in Hunan Province, Hunan, China

**Keywords:** Predictive markers, Hepatitis

## Abstract

HBV-miR-3 is encoded by HBV and takes part in pathogenesis of HBV-related liver disease. Whether HBV-miR-3 has a relationship with HBV replication and is predictive of PegIFN-α treatment response is still unknown. HBV-miR-3 quantification is based on qRT-PCR. The relationship of HBV-miR-3 and HBV replication, and predictive value of HBV-miR-3 were evaluated in a cohort of 650 HBeAg positive patients from a multi-center, randomized phase III clinical trial for the study of PegIFN-a2b. HBV-miR-3 is significantly positively related to HBVDNA, HBVpgRNA, HBeAg and HBsAg at baseline and at all the different time points during PegIFN-α treatment. Both univariate regression analyses and multivariate logistic regression analyses showed HBV-miR-3 is a predictor of HBeAg seroconversion in the patients treated with PegIFN-α at weeks of 0, 12, and 24. 70.0% of patients with HBV-miR-3 < 3log at week 12 achieved HBeAg seroconversion, otherwise, with HBV-miR-3 > 6log at week 12 no patient obtained HBeAg seroconversion. Conbination of HBV-miR-3 and HBeAg is more strongly predictive of HBeAg seroconversion (83.64%) at week 12. HBV-miR-3 is new biomarker for HBV replication and positively correlated to HBV replication. HBV-miR-3 is also an early predictor of HBeAg seroconversion in the patients treated with PegIFN-α.

## Introduction

Hepatitis B virus (HBV), a major pathogenic factor resulting in acute and chronic hepatitis, liver cirrhosis, and even hepatocellular carcinoma, affects more than 250 million people worldwide, causing nearly 1 million deaths every year^1^. Currently the antiviral agents for HBV infection are classified as nucleos(t)ide analogs (NAs) or Peg-interferon-α(PegIFN-α)^2^. NAs block HBV replication by inhibiting the activity of HBV polymerase^3^. Long-time inhibition of HBV replication by NAs improves the prognosis and quality of life. NAs treatment gets low HBeAg seroconversion rate for HBeAg positive patients and rarely obtains HBsAg clearance^4^. Drug-resistant mutations and undefined treatment courses are also limitations of NAs treatment^5^. On the other hand, PegIFN-α as an antiviral, anti-proliferative, and immuno-regulatory agent is now still used in patients with HBV infection^6^, especially for HBeAg-positive patients, with advantages of a finite course, no concern of drug-resistance, a higher rate of HBeAg seroconversion^7^ and sometimes HBsAg clearance. For HBeAg-positive patients with chronic hepatitis B(CHB), it is well known that the very important and the first therapeutic object is to get HBV replication inhibited and HBeAg seroconversion, which is the solid foundation for clinical cure(HBsAg clearance) and better prognosis. The adverse effects from PegIFN-α treatment restrict its clinical use to some extent^8^. Some biomarkers have been used as predictors for PegIFN-α treatment response ^9^, which is benefit for evaluating PegIFN-α treatment personally. Though baseline or dynamics HBVpgRNA, HBeAg, HBsAg, and HBVDNA during PegIFN-α treatment have been proposed as the useful predictors for PegIFN-α rsponsess^10^, but there still needs new biomarkers for better clinical management^11^.

Previous studies showed microRNA(miRNA) as short non-protein coding RNA^12^ plays important roles during HBV infection by participating in HBV replication and pathogenesis^13^. Recently, HBV-encoded miR-3(HBV-miR-3) was found involved in virus-host interaction and HBV replicaton^14^. There are still many problems needed to be cleared, such as whether HBV-miR-3 is related to HBV replication, persistence of HBV infection or can be used as a predictor for anti-HBV treatment response, et al. In the present study, we evaluated HBV-miR-3’s predictive role for PegIFN-α treatment response in a cohort of 650 HBeAg-positive patients who randomly received 48 weeks of PegIFN α-2a or PegIFN α-2b treatment and a 24-week of follow-up.

## Patients and methods

### Statement

This trial was registered at http://clinicaltrials.govas (NCT01760122) and China Drug Trials.org (ID: TB1211IFN). The study is conducted according to Declaration of Helsinki and was approved by the Ethics Committee of the Second Xiangya Hospital. All methods were carried out in accordance with relevant guidelines and regulations.

#### Patients

The patients are enrolled in a retrospective study based on a multi-center, randomized controlled phase III clinical trial from March 2013 to July 2015. A total of 855 HBeAg-positive CHB patients were taken as candidates and were randomized to the PegIFN α-2a group (Pegasys®, Roche, Switzerland) or PegIFN α-2b (PegBeron®, Amoytop Biotechnology, China) group by a ratio of 1:2. Patients received 48 weeks of treatment (180 μg/week) and 24 weeks of follow-up. Those candidates who failed to finish the entire study and whose serum samples were unavailable were excluded, finally, 650 patients were finally qualified for this study (The patient flow chart of this study is shown in Graphical abstract. HBsAg, HBeAg, and anti-HBe were measured using serially collected serum (Roche Diagnostics, Switzerland), HBV pgRNA was extracted from patient serum samples (200 μL) using a nucleic acid extraction kit (Sansure Biotech Inc. China) which was developed based on the magnetic bead technology, and HBV DNA was detected using quantitative real-time PCR (Sansure Biotech Inc. Hunan, China).

#### Cell lines and plasmid

The human hepatoma cell HepG2 and HepG2.2.15 cell were obtained from the Cell Bank of the Chinese Academy of Sciences (Shanghai, China). These cells were cultured in Dulbecco's modified Eagle's medium (DMEM, HyClone, Shanghai, China) supplemented with 10% fetal bovine serum (FBS, Gibco, New York, USA), and 100 U/mL penicillin and streptomycin (Catalog No. ST488, Beyotime, Beijing, China) at 37 °C in a humidified atmosphere with 5% CO_2_. The cells were seeded in 6-well plates at a density of 1 × 106 cells/well. After 24 h, a concentration gradient of HBV-miR-3 agomir or negative control (NC) RNA (synthesized by GenePharma, Shanghai, China) was transfected into HepG2 cells. The pHBV1.3 plasmid was preserved by the Infectious Disease Laboratory of the Second Xiangya Hospital. Transfection of the pHBV1.3 plasmid into HepG2 cells was performed using UltraFection 3.0 (4A Biotech China) according to the manufacturer's instructions. Transfected cells were harvested at 48 h, and DNA and RNA were extracted for detection of HBVDNA and HBVpgRNA.

#### Quantification of HBV-miR-3

MicroRNAs were extracted and purified with miRcute (Cat. No. DP501, Tiangen, Beijing, China) according to the manufacturer’s instructions, and then cDNA was synthesized using a Thermo Scientific First Strand cDNA Synthesis Kit (Cat. No. K1612, Thermo Scientific, California, USA). Cel-miR-39 (Tsingke Biological Technology, Beijing, China, Cel-miR-39:5′-UCACCGGGUGUAAAUCAGCUUG-3′) was used as an internal control to monitor miRNA extraction and amplification. For the quantification of HBV-miR-3, stem-loop RT-PCR was performed, and cDNA was synthesized using specific stem-loop miRNA-specific primers (Tsingke Biological Technology, Beijing, China HBV-miR-3 RT Primer: 5′-GTCGTATCCAGTGCAG GGTCCGAGGTGCACTGGATACGACAAACGCCG-3′. Cel-miR-39 RT Primer: 5′- GTCGTATCCAGTGCAGGGTCCGAGGTATTCGCACTGGATACGACCAAGCT-3′). HBV-miR-3 and Cel-miR-39 primers were added to the PCR reaction system. (HBV-miR-3: forward: 5′-TGCGACTGGATGTGTCTG-3′. reverse: 5′-CCA GTGCAGGGTCCGAGGT-3′. Cel39: forward: 5′-TCGCCT CACC GGGTGTA AAT C-3′, reverse: 5′-GTGCAGGGTCCGAGGT-3′). Quantitative polymerase chain reaction (Q-PCR) was performed at 95 °C for 3 min, followed by 50 cycles of 95 °C for 12 s, 60 °C for 30 s, and 72 °C for 30 s in the SLAN-96P Real-time PCR system (SLAN, China). Quantitative polymerase chain reaction (Q-PCR) was performed using miRNA-sequence specific forward primers and a common stem-loop reverse universal primer for HBV-miR-3, amplification using the fluorescent probe is carried out and wavelength-specific absorption is accurately determined and discriminated by the qPCR machine. HBV-miR-3 probe: FAM-CACTGGATACGACAAACGCCGC AGA-BHQ1 Cel39: probe: Hex-CG CACTGGATACGACCAAGCTGATT-BHQ1. A series of synthesized HBV-miR-3 standard stock solutions (tenfold serial dilutions from 10^8^ to 10^2^ copies) was generated to establish the standard dilution curve of the qPCR. The amplification curves for the validation of HBV-miR-3 is shown in Fig. S1A and B. Copy number was transformed according to the equation: copy number/mL = 6.02 × 10^23^ (copies/mol) × concentration (g/mL)/MW (g/mol). HBV DNA and HBV pgRNA in the samples revealed no significant differences in HBV-miR-3 expression, regardless of whether the samples was treated with DNase I or purified using column filtration (*p* > 0.05, Table S1).

#### Statistical analyses

Categorical and continuous variables were compared by the Fisher exact and T-test, respectively. Odds ratios (ORs) and 95% confidence intervals (CIs) were estimated from conditional logistic regressions. Logistic regression analyses were used to identify the factors associated with treatment responses. The Spearman correlation coefficient is used to quantify the correlation between continuous biomarkers. Sensitivity, specificity, PPV, and NPV for predicting HBeAg seroconversion were determined. Subsequently, the predictive power measure is based on the AUC scale.

### Ethics approval and consent to participate

The research was approved by the Ethics Committee of the Second Xiangya Hospital. All the patients enrolled in this study had gaven a written informed consent.

## Results

### Baseline demographic profiles and characteristics

221 patients received PegIFN α-2a treatment and 429 patients received PegIFN α-2b treatment. There was no significant difference in HBeAg seroconversion between PegIFN α-2a or PegIFN α-2b treatment groups. Baseline factors that affect HBeAg seroconversion include age, body weight, ALT, HBV genotype B or C, HBsAg, HBeAg, and HBV-miR-3. The details are shown in Table 1.

**Table 1:** Baseline demographic profiles and characteristics related to PegIFN response.

	All patients (*n* = 650)	SR (*n* = 188)	Non-SR (*n* = 462)	*p* value
Age (years)	28.16 ± 0.26	26.65 ± 0.65	28.82 ± 0.32	0.000
Male gender	469	132 (70.2%)	340 (73.60%)	0.370
Body weight	63.77 ± 0.45	61.78 ± 0.80	64.50 ± 0.53	0.005
ALT (IU/mL)	191.96 ± 5.43	209.6 ± 10.03	184.72 ± 6.43	0.037
HBV genotypes				
B/ C	242/402	92/95	147/310	0.000
D	6	1	5	–
HBV-miR-3#	5.77 ± 0.04	5.41 ± 0.09	5.91 ± 0.05	0.000
HBV pgRNA#	6.13 ± 0.07	5.9 ± 0.13	6.2 ± 0.07	0.085
HBV DNA^#^	7.94 ± 0.03	7.85 ± 0.05	8.0 ± 0.05	0.069
HBsAg_#_	4.27 ± 0.02	4.16 ± 0.04	4.32 ± 0.03	0.001
HBeAg^#^	3.02 ± 0.02	2.86 ± 0.04	3.08 ± 0.03	0.000
PegIFN-α 2a/2b	221/429	57/131	164/298	0.207

*SR* HBeAg seroconversion; *Non-SR* Non-HBeAg seroconversion.

^#^ The log10 transformation was performed prior to analysis. Biomarker units are measured by log10 copies/mL for HBV-miR-3 and HBV pgRNA, log10 IU/mL for HBV DNA, log10 IU/mL for HBsAg, and log10 COI for HBeAg.

### HBV-miR-3 is positively related to HBV replication

**Figure 1:**
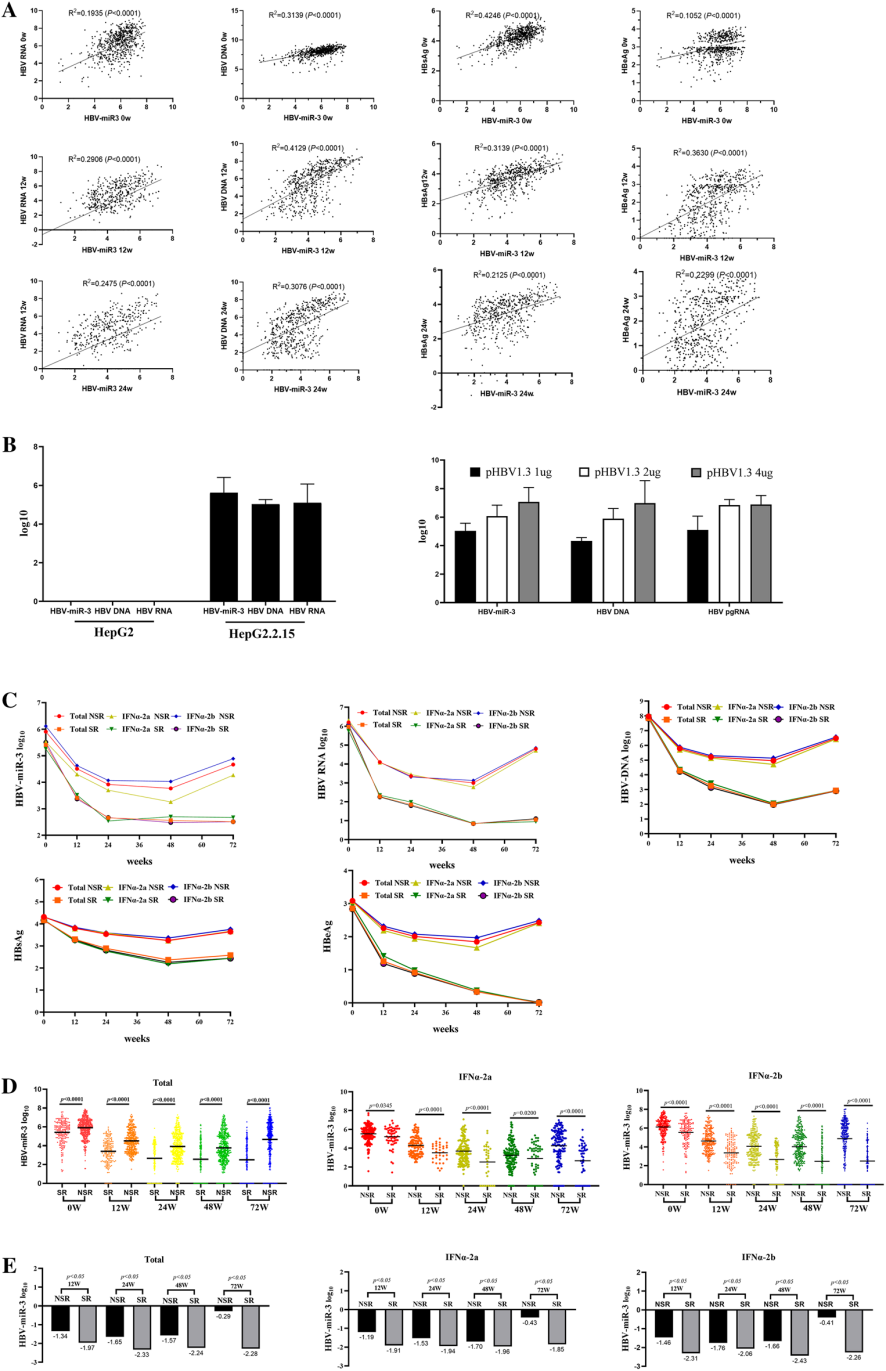
HBV biomarkers dynamic changes between the HBeAg seroconversion group and the Non-HBeAg seroconversion group. (**A**) Spearman correlation coefficient in the pairwise correlations between HBV-miR-3, HBV RNA, HBV DNA, HBsAg, and HBeAg at different time-points. (**B**) Relationship Between HBV-miR-3 and HBV Replication: HBV-miR-3, HBV DNA, and HBV pgRNA in HepG2 versus HepG2.2.15 cells and HepG2 cells versus HepG2 cells transfected with pHBV1.3 plasmid. (**C**) Dynamic changes of HBV-miR-3, HBV RNA, HBV DNA and HBeAg during PegIFN treatment. (**D**) Differences in HBV-miR-3 between the HBeAg seroconversion group and the Non-HBeAg seroconversion group at different time-points. (**E**) HBV-miR-threefold changes at 12, 24, and 36 weeks to baseline in HBeAg seroconversion and non-seroconversion groups.

Previously, HBV-miR-3 was reported as a specific microRNA encoded during HBV replication^15^, whether it is related to HBV replication needs to be evaluated. In this study, we explored the relationship between HBV-miR-3 and HBV replication-related markers, such as HBV DNA, HBV pgRNA, HBeAg and HBsAg. Our results showed in this cohort of 650 HBeAg positive chronic HBV infection patients, HBV-miR-3 is significantly positively related to HBV DNA, HBV pgRNA, HBsAg and HBeAg at baseline and all the time-points during PegIFN α treatment, indicating HBV-miR-3 is closely related to HBV replication (Fig. 1A). For further evidence the relationship between HBV-miR-3 and HBV replication. We added an in vitro experiment in which HBV-miR-3**,** HBV DNA, and HBV pgRNA were evaluated in HepG2 and HepG2.2.15 cells. The result showed only in HepG2.2.15 cell HBV-miR-3, HBV DNA, and HBV pgRNA were detected. pHBV1.3 plasmid which expressing the full HBV pgRNA was transfected into HepG2 cells with an increasing doses, HBV-miR-3, HBV DNA, and HBV pgRNA were detected and all positively correlated to the transfected pHBV1.3 doses. These experimental data indicates HBV-miR-3 level is directly related to HBV replication (Fig. 1B).

### HBV-miR-3 in HBeAg seroconversion patients maintained decreasing during PegIFN-α treatment

In all groups of patients who received PegIFN-α treatment, whether PegIFN-α2a or PegIFN-α2b, whether HBeAg seroconversion or non-HBeAg seroconversion, HBV-miR-3 decreased continuously during PegIFN α treatment. Further analysis revealed that in the HBeAg seroconversion group, HBV-miR-3 was significantly lower than non-HBeAg seroconversion group at all the time-points during the treatment (*p* < *0.0001*) and continued to decrease until week 72 (Fig. 1C,D,E). In PegIFN-2a and PegIFN-2b groups, similar dynamic changes in HBV-miR-3 were observed at all treatment time-points (*p* > *0.05*).

### HBV-miR-3 is an independent predictor of HBeAg seroconversion

Univariate regression analyses revealed that HBV-miR-3, HBV pgRNA, HBV DNA, HBsAg, and HBeAg were all significantly associated with HBeAg seroconversion at weeks 0, 12 and 24. Multivariate logistic regression analysis showed HBV-miR-3 and HBeAg were the two independent predictors for HBeAg seroconversion at weeks 0, 12 and 24 (Table 2). Whether in PegIFN-α2a or PegIFN-α2b treatment groups, HBV-miR-3 serves as an early predictor of HBeAg seroconversion at week 12 (Tables S2, S3). These results indicate that HBV-miR-3 was strongly predictive of HBeAg seroconversion in patients treated with PegIFN-α.

**Table 2:** Logistic regression analyses of predictors for HBeAg seroconversion.

	Univariate analyses			Multivariate analyses		
	OR	(95% CI)	*p*-value	OR	(95% CI)	*p*-value
0 W						
Male gender	1.186	0.817–1.722	0.370	0.833	0.502–1.383	0.480
Genotypes	0.465	0.331–0.653	0.000	0.475	0.324–0.696	0.000
Age	0.944	0.917–0.972	0.000	0.931	0.900–0.963	0.000
Body weight	0.978	0.963–0.994	0.006	0.993	0.972–1.014	0.512
ALT	1.001	1.000–1.002	0.040	1.002	1.000–1.003	0.011
HBV-miR-3	0.686	0.590–0.798	0.000	0.641	0.511–0.805	0.000
HBV pgRNA	0.408	0.832–1.011	0.000	1.004	0.885–1.139	0.951
HBV DNA	0.810	0.657–1.017	0.050	1.265	0.860–1.861	0.233
HBsAg	0.583	0.424–0.802	0.001	1.204	0.684–2.118	0.520
HBeAg	0.475	0.348–0.649	0.000	0.451	0.304–0.668	0.000
12W						
Male gender	1.186	0.817 to1.722	0.370	1.224	0.700–2.142	0.478
Genotypes	0.465	0.331–0.653	0.000	0.514	0.332–0.796	0.003
Age	0.944	0.917–0.972	0.000	0.938	0.904–0.972	0.000
Body weight	0.980	0.965–0.995	0.010	0.991	0.967–1.015	0.443
ALT	0.999	0.997–1.001	0.212	1.002	0.999–1.004	0.140
HBV-miR-3	0.440	0.365–0.531	0.000	0.548	0.431–0.697	0.000
HBV pgRNA	0.720	0.664–0.781	0.000	0.012	0.738–0.963	0.012
HBV DNA	0.642	0.578–0.712	0.000	1.202	0.940–1.536	0.143
HBsAg	0.421	0.326–0.545	0.000	1.518	1.006–2.290	0.047
HBeAg	0.402	0.332–0.486	0.000	0.419	0.298–0.590	0.000
24W						
Male gender	1.186	0.817–1.722	0.370	1.077	0.610–1.902	0.799
Genotypes	0.465	0.331–0.653	0.000	0.667	0.424–1.048	0.079
Age	0.944	0.917–0.972	0.000	0.939	0.905–0.974	0.001
Body weight	0.982	0.966–0.997	0.023	1.002	0.978–1.027	0.862
ALT	0.996	0.993–0.999	0.005	0.998	0.994–1.002	0.260
HBV-miR-3	0.573	0.501–0.655	0.000	0.692	0.585–0.819	0.000
HBV pgRNA	0.781	0.722–0.844	0.000	1.136	0.995–1.298	0.060
HBV DNA	0.641	0.581–0.706	0.000	0.917	0.735–1.144	0.442
HBsAg	0.512	0.420–0.624	0.000	1.162	0.861–1.568	0.326
HBeAg	0.322	0.257–0.403	0.000	0.356	0.250–0.509	0.000

### HBV-miR-3 performs better in PPV for HBeAg seroconversion

**Figure 2:**
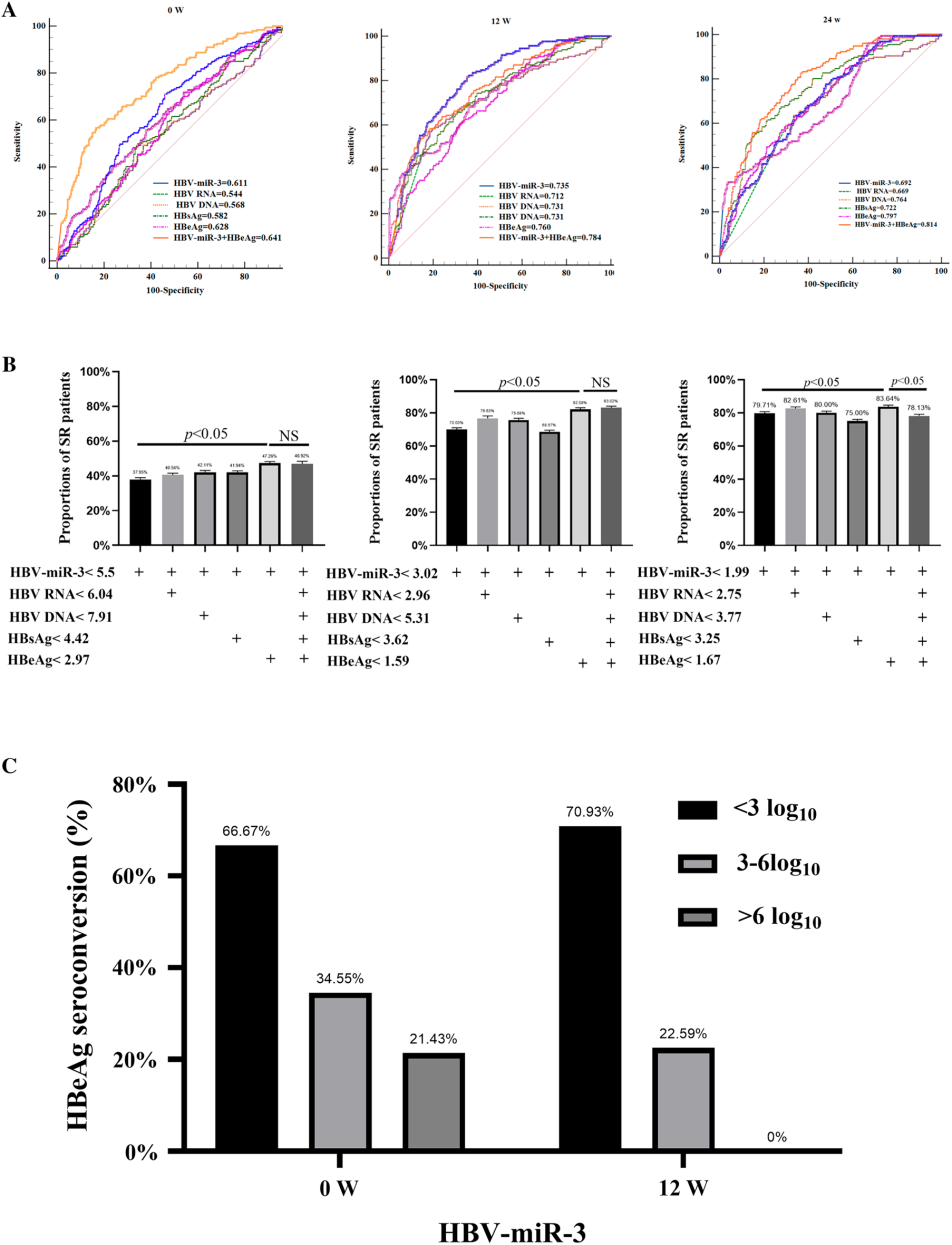
HBV-miR-3 is an early predictor for HBeAg seroconversion in the HBeAg-positive patients treated with PegIFN-α. (**A**) AUC values of HBV-miR3 at weeks 0 and 12. (**B**)Predictive performance of biomarker cut-off values and their combined performance. (**C**)Substrate analysis of HBV-miR3 level for the PegIFN-a response.

Receiver operating characteristic (ROC) curve analysis indicated that HBV-miR-3 performed better than HBV pgRNA, HBV DNA, and HBsAg at early stages, and the combination of HBV-miR-3 and HBeAg performed best at week 12 (Fig. 2A). All the markers at baseline showed similar performance in PPV. HBV-miR-3 showed 70.00% and 79.71% of PPVs at week 12 or 24, better than HBV pgRNA, HBV DNA, and HBsAg, and even HBeAg (Table 3). We screened all five available factors (HBV-miR-3, HBV pgRNA, HBV DNA, HBsAg, and HBeAg) to identify the most effective combination. The results showed that the combination of HBV-miR-3 and HBeAg had the largest area under the ROC curve (Fig. 2A), and the best predictive effect in the early stages (Fig. 2B). For more convenient application in the clinical practice, we arbitrarily grouped the patients into three groups (HBV-miR-3 ≤ 3 log10 copies/ml, 3 < HBV-miR-3 ≤ 6 log10 copies/ml and HBV-miR-3 > 6 log10 copies/ml). Seventy percent of patients whose HBV-miR-3 ≤ 3 log10 copies/ml at week 12 achieved HBeAg seroconversion, but when HBV-miR-3 is still more than 6 log10 copies/ml at week 12, no patient obtained HBeAg seroconversion (Fig. 2C).

**Table 3:** Analysis of PPV and NPV of patients with HBeAg seroconversion.

	Cutoff(log10)	Positive predictive value (PPV) (%)	Negative predictive value (NPV) (%)	Accuracy (%)
0W				
HBV-miR-3	≤ 5.50	37.95	75.82	62.76
HBV pgRNA	≤ 6.04	35.50	75.45	70.13
HBV DNA	≤ 7.91	36.76	76.07	60.77
HBsAg	≤ 4.42	35.21	78.64	54.92
HBeAg	≤ 2.97	38.73	82.24	59.08
12W				
HBV-miR-3	≤ 3.02	70.00	79.66	78.43
HBV pgRNA	≤ 2.83	54.07	82.97	74.53
HBV DNA	≤ 5.31	45.00	83.95	68.08
HBsAg	≤ 3.62	41.53	81.30	65.37
HBeAg	≤ 1.59	50.48	84.51	72.50
24W				
HBV-miR-3	≤ 1.99	79.71	79.92	79.90
HBV pgRNA	≤ 2.75	39.85	83.33	83.55
HBV DNA	≤ 3.77	47.56	85.60	71.33
HBsAg	≤ 3.25	41.52	81.87	65.68
HBeAg	≤ 1.67	45.42	90.88	70.48

### The role played by HBV-miR-3 in the prediction of HBsAg

In the present study, only 17 patients achieved HBsAg loss, so it is hard to evaluate the HBV-miR-3 prediction of HBsAg loss. We set HBsAg < 100 IU/ml (77 patients) at week 72 as the endpoint for analysis (Table S4). univariate logistic regression analysis (Table S5) showed HBV-miR-3, HBV pgRNA, HBV DNA, and HBsAg were the predictors for HBsAg < 100 IU/mL at week 12, but HBsAg was the independent predictor by multivariate logistic regression analysis (Fig. S2A,B).

## Discussion

PegIFN-α is an essential anti-HBV medication for patients with chronic hepatitis B, with higher HBeAg seroconversion and the potential for HBsAg loss comparable to NAs^16^. Additionally, PegIFN-α therapy benefits some specific patient populations, including for shorter treatment duration, lower risk of drug resistance, and sustained off-therapy response^17^. Some patients are intolerable PegIFN-α therapy due to the side effects, leading to discontinuation of treatment^18^. Predictors for PegIFN-α treatment response can help patients and physicians make decision regarding treatment continuation or timely discontinuation. Some classic HBV biomarkers, such as HBV DNA, HBsAg, and HBeAg, not only serve as indicators of HBV replication but also hold significant value for predicting the response to PegIFN treatment^19^. Other factors such as HBV genotype, age, gender, and ALT levels also influence PegIFN-α treatment outcomes^20^. Recently, some new HBV biomarkers such as HBcrAg^21^, HBV pgRNA^22^ and quantitative hepatitis B core antibody (qAnti-HBc) were proven useful in monitoring HBV replication and response to PegIFN-α treatment. HBcrAg consists of three proteins, namely HBcAg, HBeAg, and p22Cr (22 kDa precursor protein), encoded by the pre-C/C region gene of HBV^23^, with the same 149 amino acid sequence. It has been reported that HBcrAg reflects the amount and transcriptional activity of cccDNA in hepatocytes^24^. During PegIFN-α treatment, HBcrAg decreased more strongly in response patients. Lower HBcrAg and higher HBsAb levels at End-of-treatment are associated with immune responses after PegIFN-α based therapy^16^, but are limited by the problem of mutations in the pre-C/C region gene of HBV. Serum HBV pgRNA is one of the hot spots of research in recent years^25^. As a new serological marker, it is a virus-like particle containing HBV pregenomic RNA (HBV pgRNA) and its splice variants. HBV pgRNA is formed by direct transcription of intrahepatic cccDNA^26^, therefore, serum HBV pgRNA can also reflect the amount and activity of intrahepatic cccDNA to some extent, which is useful for reflecting the response to antiviral therapy. Compared to HBVDNA, and HBeAg, HBV pgRNA at week 12 is a more effective monitor of HBeAg seroconversion in HBeAg-positive patients treated with Pegylated interferons, indicating HBV pgRNA is an early predictor for PegIFN-α reponse^27^. Recently, anti-HBc quantification especially at baseline, may act as a predictor of PegIFN-α response. Patients with baseline qAnti-HBc levels ≥ 30,000 IU/mL had significantly higher response rates, more HBV DNA suppression, and better hepatitis control in PegIFN-α treatment^28^, but the qAnti-HBc during PegIFN treatment maintained unchanged, and this phenomenon devalued qAnti-HBc monitoring significance of the PegIFN-α response.

HBV-miR-3, which is encoded from nucleotides 373 to 393 in the HBV genome, is derived from three HBV transcripts: PreC, PreS1, and PreS2 mRNAs^14^. Several studies have suggested that HBV-miR-3 plays a role in the development of HBV-associated hepatocellular carcinoma (HCC) by targeting PTEN^15^, and PPM1A16^29^. Furthermore, HBV DNA and HBV-miR-3 expression show a positive correlation in CHB patients^14^. It is unknown whether HBV-miR-3 can be used as a biomarker for HBV replication or as a predictor of interferon response. In this present study with a cohort of 650 patients who received treatment with either PegIFN-α 2a or 2b. our results showed a positive correlation between HBV-miR-3 and reported HBV replication markers including HBV DNA, HBsAg, HBeAg, and HBV pgRNA, both at baseline and at different time-points during PegIFN-α treatment. Our experimental results show that HBV-miR-3 is closely related with HBV replication. HBV-miR-3 expression is depend on the doses of pHBV1.3 plasmid transfected into HepG2 cells, which is closely correlated to HBV DNA and HBV pgRNA. Our clinical and experimental data indicated that HBV-miR-3 is closely related to HBV replication, which is consist with previous studies^30^.

From our present study and previous reports, it is known that HBV-miR-3 is closely related to HBV replication. However, it is not known whether HBV-miR-3 can be used as a predictor for PegIFN-α response. There is rarely report on HBV-miR-3’s prediction on anti-HBV therapy response. In this present study with a cohort of 650 HBeAg positive CHB patients treated with PegIFN-a, we found HBV-miR-3 is greatly decreased during treatment especially at early stage. HBV-miR-3 decreasing is consistent with HBV DNA, HBV pgRNA, HBsAg and HBeAg. Compared to non-HBeAg seroconversion group, HBeAg seroconversion group showed a greater fold decreasing. We also found HBV-miR-3 is an early (12W) independent predictor for HBeAg seroconversion, whether by univariate and multivariate regression analyses. Finally, HBV-miR-3 decreased to or less than 3 logs at 12W, 70% of patients will get to HBeAg seroconversion, but still with 6 logs or more at 12W no patients will become HBeAg seroconversion. These results indicated HBV-miR-3 may be an early predictor for HBeAg seroconversion in HBeAg positive patients with PegIFN-a treatment. We also explored the role of HBV-miR-3 in predicting HBsAg levels. HBV-miR-3 showed a positive correlation with HBsAg levels at baseline and during treatment. In the HBsAg < 100 IU/mL group, HBV-miR-3 levels were significantly lower compared to the group with HBsAg ≥ 100 IU/mL. However, the results from multivariate logistic analysis indicate that HBV-miR-3 is not an independent predictor for HBsAg < 100 IU/mL, with only HBsAg itself serving as the independent predictor during treatment.

Above all, HBV-miR-3 can be used as a predictor for HBeAg seroconversion in HBeAg positive patients with PegIFN-αtreatment, but our study had several limitations. First, there is no commercially available diagnostic agent for measuring HBV-miR-3. Second, our study included HBeAg-positive patients only and the results were not applied to HBeAg-negative patients, HBV-miR-3’s role in prediction of HBsAg loss is still needed to be elucidated. Finally, our study was a retrospective study, more studies, especially prospective studies are needed.

The patients are enrolled in a retrospective study based on a multi-center, randomized controlled phase III clinical trial from March 2013 to July 2015. A total of 855 HBeAg-positive CHB patients were taken as candidates and were randomized to the PegIFN α-2a group (Pegasys®, Roche, Switzerland) or PegIFN α-2b (PegBeron®, Amoytop Biotechnology, China) group by a ratio of 1:2. Patients received 48 weeks of treatment (180 μg/week) and 24 weeks of follow-up. Those candidates who failed to finish the entire study and whose serum samples were unavailable were excluded, finally, 650 patients were finally qualified for this study (The patient flow chart of this study is shown in Graphical abstract. HBsAg, HBeAg, and anti-HBe were measured using serially collected serum (Roche Diagnostics, Switzerland), HBV pgRNA was extracted from patient serum samples (200 μL) using a nucleic acid extraction kit (Sansure Biotech Inc. China) which was developed based on the magnetic bead technology, and HBV DNA was quantified with Roche Diagnostics Cobas® Amplicor HBV Test, Version 2.0 (Roche Diagnostics, Switzerland). This trial was registered at http://clinicaltrials.govas (NCT01760122) and China Drug Trials.org (ID: TB1211IFN).

### Supplementary Information

Supplementary Information.

## Data Availability

The data that support the findings of this study are available in https://clinicaltrials.gov/study/NCT01760122?id=NCT01760122&rank=1. This trial was registered at http://clinicaltrials.govas (NCT01760122).

## Abbreviations

ALT: Alanine aminotransferase
anti-HBe: Hepatitis B e antibody
anti-HBs: Hepatitis B s antibody
AUC: Area under the receiver operating characteristic curve
cccDNA: Covalently closed circular DNA
HBeAg: Hepatitis B e antigen
HBsAg: Hepatitis B s antigen
HL: HBsAg loss
NA: Nucleos(t)ide analogue
NPV: Negative predictive value
OR: Odds-ratio
PegIFNα-2a: Pegylated interferon alfa-2a
PegIFNα-2b: Pegylated interferon alfa-2b
PPV: Positive predictive value
ROC: Receiver operating characteristic curve
SR: Serological response
