# Supplementary material for: HBV-miR-3 is closely related to HBV replication and strongly predictive of HBeAg seroconversion in PegIFN-α treated patients
Source: Sci Rep. 2024 Jan 17;14:1502. doi: 10.1038/s41598-024-52060-0 (PMC10794194; doi:10.1038/s41598-024-52060-0)
Supplement: Supplementary file 1 — Supplementary Information. [file 41598_2024_52060_MOESM1_ESM.docx]

**HBV-miR-3 is related to HBV replication and predictive of HBeAg seroconversion in PegIFN-α treated patients**

**Author:** Zhenyu Xu^1^, Yun Xu^1^, Zhenyu Wu^1^, Sujuan wang^1^, Min Zhang^1*^, Yongfang Jiang^1*^, Guiqiang Wang ^2 *^, Guozhong Gong^1*^

**Supplementary data**

[Supplementary Methods 2](#_Toc21050)

[Supplementary Figures 4](#_Toc27134)

[Supplementary Tables 6](#_Toc5592)

## Supplementary Methods

HBV-miR-3 standard

A series of HBV-miR-3 standard stock solution (10-fold serial dilutions from 10^8^copies to 10^2^ copies) was generated according to the formula to establish the dilution curve of the qPCR standard. Formula: Copy number was determined according to the equation: copy number/mL = 6.02×10^23^ (copies/mol)×concentration (g/mL)/MW (g/mol). HBV-miR-3 template from the literature^30^: TGCGACTGGATGTGTCTGCGGCGTTTGTCGTATCCAGTGCACCTCGGACCCTGCACTGG


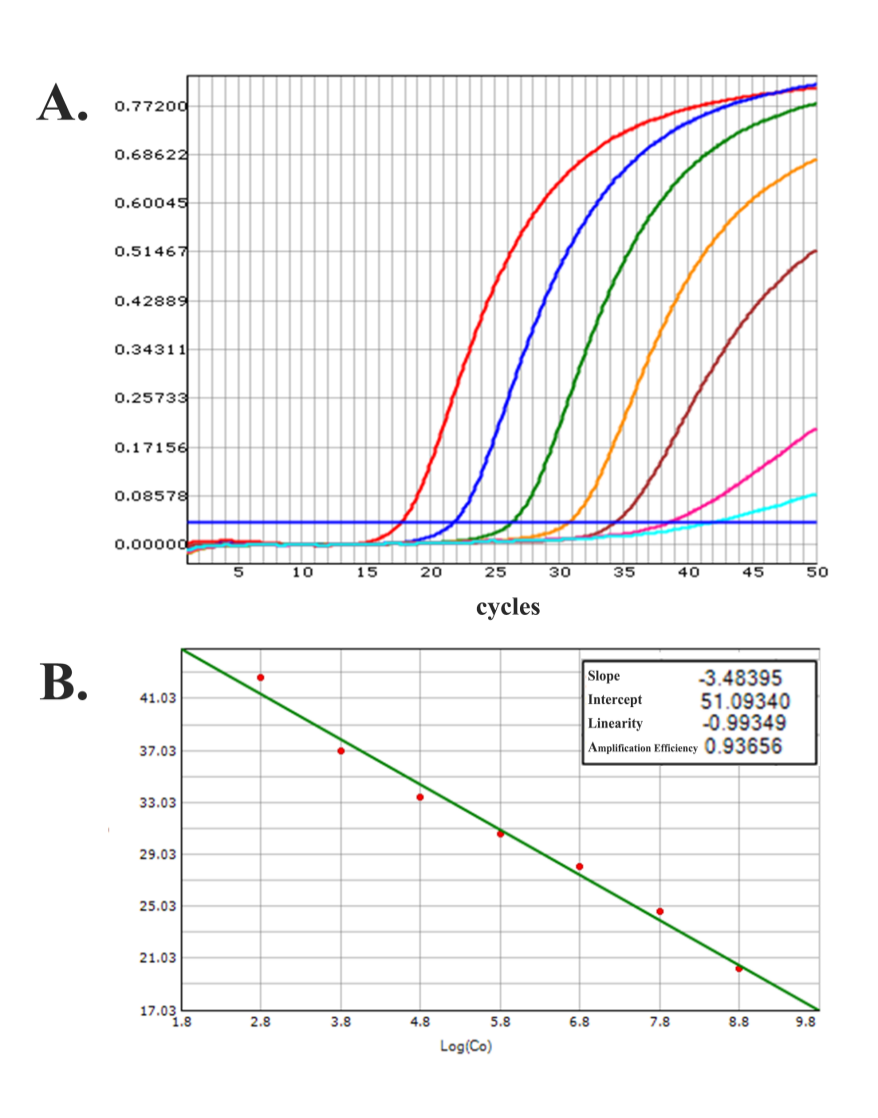


Figure S1.(A) Amplification curves of HBV-miR-3 that were measured by the replication cycles versus delta Rn. Amplification curves show the results of HBV-miR-3 at the concentrations from 6×10^2^ to 6×10^8^ copies/mL. (B) Correlations between the cycle threshold (Ct) and the concentration of HBV-miR-3. The x-axis indicates the log10 values of HBV-miR-3 concentrations from 6×10^2^ copies/mL to 6×10^8^ copies/mL, while the y-axis shows the cycle threshold. The correlation coefficient was -3.48395 with R^2^=0.99349.

Table S1 Detection of HBV-miR-3 in samples harboring the high concentrations of

HBV DNA treated with or without DNase I or before and after passing the column.

| Absence of DNase I | | Treated with DNase I | | p-value  (a) vs. (b) |
| --- | --- | --- | --- | --- |
| DNA | HBV-miR-3(a) | DNA | HBV-miR-3 (b) |  |
| 3 | 2.78±0.09* | <LOD | 2.75±0.01 | 0.2526 |
| 4 | 3.90±0.02 | <LOD | 3.82±0.06 | 0.9526 |
| 5 | 4.50±0.03 | <LOD | 4.21±0.02 | 0.1756 |
| before | | after | | p-value  (a) vs. (b) |
| RNA | HBV-miR-3(a) | pgRNA | HBV-miR-3 (b) |  |
| 3.21±0.02* | 2.75±0.08 | <LOD | 2.79±0.11 | 0.1294 |
| 3.02±0.09* | 2.80±0.02 | <LOD | 2.78±0.06 | 0.2526 |
| 2.78±0.09* | 2.76±0.03 | <LOD | 2.69±0.02 | 0.1756 |

*: Mean ± standard deviation (n=3); LOD: Limit of detection for HBV DNA is 20

IU/mL. HBV DNA and HBV-miR-3 were quantified by log10 IU/mL and log10 copies/mL, respectively. *: Mean ± standard deviation (n=3); LOD: Limit of detection for HBV DNA is 300 copies/mL. HBV pgRNA and HBV-miR-3 were quantified by log10 copies/mL, respectively.

## Supplementary Figures


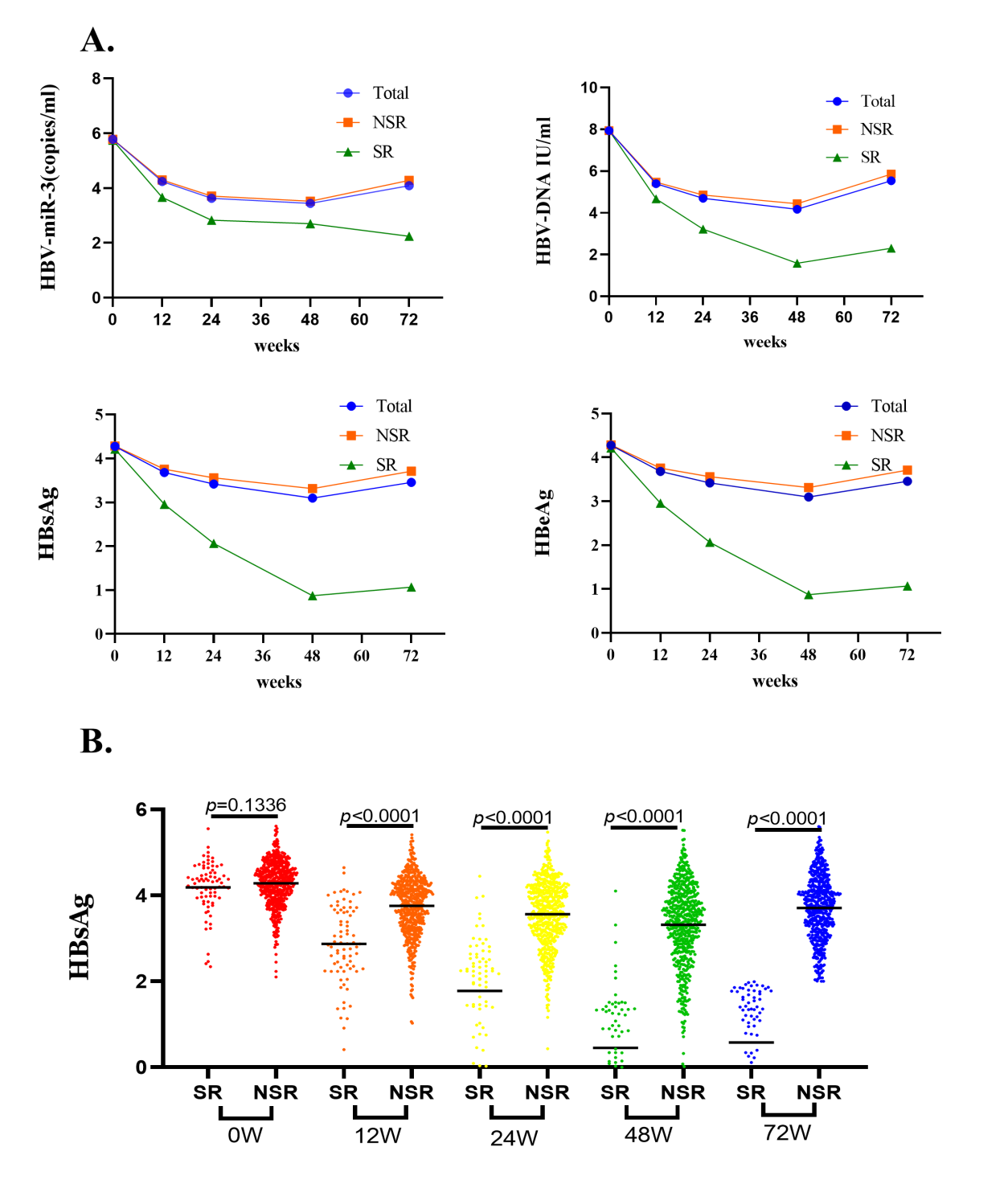


Figure S2

Figure S2 : HBV biomarker change between HBsAg≦100 IU/ml group and HBsAg>100 IU/ml group. A: Dynamic changes of HBV-miR3, HBV DNA, HBsAg, and HBeAg. B: Differences in HBV-miR3 between HBsAg≦100 IU/ml group and HBsAg>100 IU/ml group at different time.

## Supplementary Tables

Table S2 Logistic regression analyses of predictors for HBeAg seroconversion in the PegIFN α-2a treatment group

|  | Univariate Analyses | | |  | Multivariate Analyses | | |
| --- | --- | --- | --- | --- | --- | --- | --- |
|  | OR | (95% CI) | p-value |  | OR | (95% CI) | p-value |
| 0 W |  |  |  |  |  |  |  |
| Male gender | 1.124 | 0.577 to 2.189 | 0.731 |  | 1.350 | 0.529 to 3.441 | 0.530 |
| Genotypes | 0.377 | 0.204 to 0.697 | 0.002 |  | 0.406 | 0.206 to 0.8 | 0.009 |
| Age | 0.913 | 0.862 to 0.968 | 0.002 |  | 0.905 | 0.847 to 0.967 | 0.003 |
| Body weight | 0.982 | 0.954 to 1.011 | 0.215 |  | 0.984 | 0.946 to 1.025 | 0.445 |
| ALT | 1.000 | 0.998 to 1.002 | 0.983 |  | 1.000 | 0.997 to 1.003 | 0.999 |
| HBV-miR-3 | 0.719 | 0.527 to 0.982 | 0.038 |  | 0.701 | 0.434 to 1.135 | 0.148 |
| HBV RNA | 0.846 | 0.72 to 0.994 | 0.042 |  | 0.870 | 0.715 to 1.058 | 0.162 |
| HBV DNA | 0.958 | 0.644 to 1.424 | 0.832 |  | 1.921 | 0.873 to 4.226 | 0.105 |
| HBsAg | 0.598 | 0.335 to 1.069 | 0.083 |  | 0.521 | 0.180 to 1.513 | 0.231 |
| HBeAg | 0.672 | 0.389 to 1.161 | 0.154 |  | 0.752 | 0.354 to 1.597 | 0.458 |
| 12W |  |  |  |  |  |  |  |
| Male gender | 1.124 | 0.577 to 2.189 | 0.731 |  | 1.701 | 0.508 to 5.701 | 0.389 |
| Genotypes | 0.377 | 0.204 to 0.697 | 0.002 |  | 0.438 | 0.184 to 1.043 | 0.062 |
| Age | 0.913 | 0.862 to 0.968 | 0.002 |  | 0.903 | 0.829 to 0.984 | 0.020 |
| Body weight | 0.984 | 0.957 to 1.012 | 0.253 |  | 0.983 | 0.936 to 1.003 | 0.501 |
| ALT | 0.998 | 0.994 to 1.002 | 0.368 |  | 1.004 | 1.000 to 1.009 | 0.059 |
| HBV-miR-3 | 0.416 | 0.264 to 0.657 | 0.000 |  | 0.568 | 0.32 to 0.970 | 0.049 |
| HBV RNA | 0.731 | 0.638 to 0.836 | 0.000 |  | 0.783 | 0.595 to 1.030 | 0.080 |
| HBV DNA | 0.675 | 0.568 to 0.803 | 0.000 |  | 0.767 | 0.432 to 1.363 | 0.366 |
| HBsAg | 0.358 | 0.23 to 0.557 | 0.000 |  | 1.689 | 0.625 to 4.565 | 0.302 |
| HBeAg | 0.495 | 0.364 to 0.674 | 0.000 |  | 0.937 | 0.455 to 1.930 | 0.860 |
| 24W |  |  |  |  |  |  |  |
| Male gender | 1.124 | 0.577 to 2.189 | 0.731 |  | 1.607 | 0.450 to 5.747 | 0.465 |
| Genotypes | 0.377 | 0.204 to 0.697 | 0.002 |  | 0.565 | 0.227 to 1.407 | 0.220 |
| Age | 0.913 | 0.862 to 0.968 | 0.002 |  | 0.913 | 0.836 to 0.997 | 0.043 |
| Body weight | 0.380 | 0.960 to 1.016 | 0.380 |  | 1.010 | 0.957 to 1.066 | 0.717 |
| ALT | 0.995 | 0.990 to 1.000 | 0.073 |  | 0.998 | 0.989 to 1.006 | 0.575 |
| HBV-miR-3 | 0.557 | 0.413 to 0.753 | 0.000 |  | 0.567 | 0.387 to 0.832 | 0.004 |
| HBV RNA | 0.791 | 0.697 to 0.899 | 0.000 |  | 1.231 | 0.917 to 1.654 | 0.166 |
| HBV DNA | 0.684 | 0.585 to 0.800 | 0.000 |  | 1.004 | 0.623 to 1.617 | 0.987 |
| HBsAg | 0.427 | 0.302 to 0.605 | 0.000 |  | 0.701 | 0.389 to 1.264 | 0.238 |
| HBeAg | 0.377 | 0.263 to 0.540 | 0.000 |  | 0.290 | 0.192 to 0.914 | 0.029 |

Table S3 Logistic regression analyses of predictors for HBeAg seroconversion in the PegIFN α-2b treatment group

|  | Univariate Analyses | | |  | Multivariate Analyses | | |
| --- | --- | --- | --- | --- | --- | --- | --- |
|  | OR | (95% CI) | p-value |  | OR | (95% CI) | p-value |
| 0 W |  |  |  |  |  |  |  |
| Male gender | 0.725 | 0.461 to1.141 | 0.165 |  | 0.727 | 0.390 to 1.355 | 0.315 |
| Genotypes | 0.509 | 0.338 to 0.766 | 0.001 |  | 0.463 | 0.283 to 0.755 | 0.000 |
| Age | 0.956 | 0.925 to 0.989 | 0.009 |  | 0.946 | 0.909 to 0.985 | 0.000 |
| Body weight | 0.982 | 0.954 to 1.011 | 0.215 |  | 0.991 | 0.966 to 1.017 | 0.492 |
| ALT | 1 | 0.998 to 1.002 | 0.983 |  | 1.002 | 1.000 to 1.005 | 0.001 |
| HBV-miR-3 | 0.534 | 0.408 to 0.698 | 0 |  | 0.477 | 0.345 to 0.659 | 0.000 |
| HBV RNA | 1.206 | 1.019 to 1.427 | 0.029 |  | 1.199 | 1.004 to 1.431 | 0.045 |
| HBV DNA | 1.149 | 0.759 to 1.738 | 0.511 |  | 0.876 | 0.561 to 1.368 | 0.560 |
| HBsAg | 1.414 | 0.777 to 2.574 | 0.256 |  | 1.717 | 0.878 to 3.358 | 0.114 |
| HBeAg | 0.464 | 0.3 to 0.718 | 0.001 |  | 0.411 | 0.254 to 0.664 | 0.000 |
| 12W |  |  |  |  |  |  |  |
| Male gender | 0.725 | 0.461 to1.141 | 0.165 |  | 1.217 | 0.634 to 2.336 | 0.554 |
| Genotypes | 0.509 | 0.338 to 0.766 | 0.001 |  | 0.468 | 0.278 to 0.789 | 0.004 |
| Age | 0.956 | 0.925 to 0.989 | 0.009 |  | 0.950 | 0.912 to 0.989 | 0.013 |
| Body weight | 0.975 | 0.956 to 0.994 | 0.009 |  | 0.991 | 0.964 to 1.019 | 0.525 |
| ALT | 0.999 | 0.997 to 1.002 | 0.470 |  | 1.002 | 0.999 to 1.004 | 0.226 |
| HBV-miR-3 | 0.431 | 0.350 to 0.531 | 0.000 |  | 0.521 | 0.396 to 0.688 | 0.000 |
| HBV RNA | 0.719 | 0.656 to 0.789 | 0.000 |  | 0.871 | 0.746 to 1.017 | 0.080 |
| HBV DNA | 0.631 | 0.576 to 0.710 | 0.000 |  | 1.294 | 0.979 to 1.711 | 0.070 |
| HBsAg | 0.376 | 0.279 to 0.508 | 0.000 |  | 1.344 | 0.845 to 2.137 | 0.212 |
| HBeAg | 0.368 | 0.295to 0.460 | 0.000 |  | 0.366 | 0.245 to 0.546 | 0.000 |
| 24W |  |  |  |  |  |  |  |
| Male gender | 0.725 | 0.461 to1.141 | 0.165 |  | 1.159 | 0.599 to 2.244 | 0.661 |
| Genotypes | 0.509 | 0.338 to 0.766 | 0.001 |  | 0.681 | 0.402 to 1.153 | 0.153 |
| Age | 0.956 | 0.925 to 0.989 | 0.009 |  | 0.954 | 0.915 to 0.995 | 0.027 |
| Body weight | 0.997 | 0.958 to 0.996 | 0.017 |  | 0.997 | 0.970 to 1.026 | 0.851 |
| ALT | 0.996 | 0.992 to 0.999 | 0.015 |  | 0.998 | 0.994 to 1.003 | 0.444 |
| HBV-miR-3 | 0.550 | 0.471 to 0.643 | 0.000 |  | 0.686 | 0.564 to 0.836 | 0.000 |
| HBV RNA | 0.774 | 0.708 to 0.846 | 0.000 |  | 1.166 | 0.998 to 1.364 | 0.054 |
| HBV DNA | 0.611 | 0.544 to 0.685 | 0.000 |  | 0.908 | 0.705 to 1.170 | 0.457 |
| HBsAg | 0.440 | 0.345 to 0.562 | 0.000 |  | 0.962 | 0.678 to 1.364 | 0.827 |
| HBeAg | 0.287 | 0.220 to 0.374 | 0.000 |  | 0.335 | 0.217 to 0.516 | 0.000 |

Table S4 Baseline analysis of patient characteristics for HBsAg <100 IU/ml

|  | HBsAg<100  (*n* = 77) | HBsAg≧100  (n = 573) | *p* value |
| --- | --- | --- | --- |
| Age (years) | 25.95 ± 0.59 | 28.46 ± 0.29 | 0.02 |
| Male gender | 48 (62.3%) | 421 (73.5%) | 0.06 |
| Body weight | 61.01 ±1.27 | 64.08 ± 0.47 | 0.02 |
| ALT (IU/mL) | 209.31 ± 16.1 | 189.55 ± 5.76 | 0.23 |
| HBV genotypes |  |  |  |
| B/C | 42/35 | 200/367 | 0.001 |
| HBV-miR-3 | 5.65 ± 0.14 | 5.78 ± 0.47 | 0.35 |
| HBV RNA# | 6.11 ± 0.21 | 6.13 ± 0.07 | 0.92 |
| HBV DNA# | 7.91 ± 0.71 | 7.94 ± 0.32 | 0.77 |
| HBsAg_#_ | 4.19 ± 0.07 | 4.28 ± 0.02 | 0.13 |
| HBeAg# | 3.10 ± 0.05 | 3.00 ± 0.02 | 0.14 |
| pegIFNα-2a/pegIFNα-2b | 27/50 | 194/379 | 0.83 |

Table S5 Univariate and Multivariate analyses for prediction of HBsAg <100IU/ml

|  | Univariate Analyses | | |  | Multivariate Analyses | | |
| --- | --- | --- | --- | --- | --- | --- | --- |
|  | OR | (95% CI) | p-value |  | OR | (95% CI) | p-value |
| 0 W |  |  |  |  |  |  |  |
| Male gender | 0.591 | 0.341 to 1.023 | 0.060 |  | 0.560 | 0.266 to 1.180 | 0.127 |
| Genotypes | 0.425 | 0.251 to 0.719 | 0.001 |  | 0.384 | 0.216 to 0.681 | 0.001 |
| Age | 0.929 | 0.884 to 0.976 | 0.004 |  | 0.934 | 0.885to 0.986 | 0.013 |
| Body weight | 0.978 | 0.954 to 1.003 | 0.084 |  | 1.001 | 0.969 to 1.034 | 0.967 |
| ALT | 1.001 | 0.954 to 1.003 | 0.216 |  | 1.002 | 1.000 to 1.004 | 0.072 |
| HBV-miR-3 | 0.968 | 0.757 to 1.239 | 0.798 |  | 1.053 | 0.728 to 1.525 | 0.783 |
| HBV RNA | 0.993 | 0.863 to 1.143 | 0.924 |  | 1.047 | 0.863 to 1.269 | 0.642 |
| HBV DNA | 1.003 | 0.706 to 1.425 | 0.988 |  | 1.103 | 0.593 to 2.051 | 0.757 |
| HBsAg | 0.786 | 0.482 to 1.283 | 0.336 |  | 0.397 | 0.168 to 0.937 | 0.035 |
| HBeAg | 1.314 | 0.808to 2.136 | 0.271 |  | 1.808 | 0.965 to 3.390 | 0.065 |
| 12W |  |  |  |  |  |  |  |
| Male gender | 0.591 | 0.341 to 1.023 | 0.060 |  | 0.565 | 0.245 to 1.305 | 0.181 |
| Genotypes | 0.425 | 0.251 to 0.719 | 0.001 |  | 1.140 | 0.306 to 1.140 | 0.117 |
| Age | 0.929 | 0.884 to 0.976 | 0.004 |  | 0.949 | 0.895 to 1.006 | 0.079 |
| Body weight | 0.976 | 0.952 to 1.002 | 0.067 |  | 1.003 | 0.996 to 1.041 | 0.894 |
| ALT | 1.001 | 0.998 to 1.004 | 0.402 |  | 1.002 | 0.998 to 1.005 | 0.289 |
| HBV-miR-3 | 0.899 | 0.807 to1.002 | 0.001 |  | 0.929 | 0.665 to 1.298 | 0.667 |
| HBV RNA | 0.870 | 0.789 to 0.960 | 0.005 |  | 1.054 | 0.866 to 1.283 | 0.597 |
| HBV DNA | 0.816 | 0.712 to 0.935 | 0.003 |  | 1.045 | 0.700 to 1.559 | 0.830 |
| HBsAg | 0.243 | 0.167 to 0.357 | 0.000 |  | 0.134 | 0.072 to 0.253 | 0.000 |
| HBeAg | 0.857 | 0.676to 1.086 | 0.202 |  | 2.059 | 1.185 to 3.579 | 0.010 |
| 24W |  |  |  |  |  |  |  |
| Male gender | 0.591 | 0.341 to 1.023 | 0.060 |  | 0.424 | 0.154 to 1.168 | 0.970 |
| Genotypes | 0.425 | 0.251 to 0.719 | 0.001 |  | 1.220 | 0.545 to 2.730 | 0.629 |
| Age | 0.929 | 0.884 to 0.976 | 0.004 |  | 0.929 | 0.859 to 1.004 | 0.063 |
| Body weight | 0.976 | 0.951 to 1.002 | 0.068 |  | 1.017 | 0.974 to 1.063 | 0.436 |
| ALT | 0.998 | 0.994 to 1.002 | 0.341 |  | 0.997 | 0.992 to 1.003 | 0.354 |
| HBV-miR-3 | 0.710 | 0.596 to 0.847 | 0.000 |  | 0.984 | 0.738 to 1.311 | 0.910 |
| HBV RNA | 0.850 | 0.777 to 0.947 | 0.002 |  | 1.422 | 1.093 to 1.851 | 0.009 |
| HBV DNA | 0.648 | 0.591 to 0.709 | 0.000 |  | 1.287 | 0.844 to 1.963 | 0.242 |
| HBsAg | 0.154 | 0.102 to 0.233 | 0.000 |  | 0.048 | 0.021 to 0.108 | 0.000 |
| HBeAg | 0.666 | 0.514 to 0.863 | 0.002 |  | 1.313 | 0.696 to 2.476 | 0.401 |
